# Health Risks of Temperature Variability on Hospital Admissions in Cape Town, 2011–2016

**DOI:** 10.3390/ijerph20021159

**Published:** 2023-01-09

**Authors:** Malebo Sephule Makunyane, Hannes Rautenbach, Neville Sweijd, Joel Botai, Janine Wichmann

**Affiliations:** 1 School of Health Systems and Public Health, Faculty of Health Sciences, University of Pretoria, Pretoria 0002, South Africa; 2 South African Weather Service, Pretoria 0001, South Africa; 3 Faculty of Natural Sciences, Akademia, Pretoria 0002, South Africa; 4 Applied Centre for Climate and Earth Systems Science, Council for Scientific and Industrial Research, Cape Town 7700, South Africa; 5 Department of Geography, Geoinformatics and Meteorology, University of Pretoria, Pretoria 0002, South Africa

**Keywords:** temperature variability, cardiovascular diseases, respiratory diseases, hospital admissions, South Africa

## Abstract

Epidemiological studies have provided compelling evidence of associations between temperature variability (TV) and health outcomes. However, such studies are limited in developing countries. This study aimed to investigate the relationship between TV and hospital admissions for cause-specific diseases in South Africa. Hospital admission data for cardiovascular diseases (CVD) and respiratory diseases (RD) were obtained from seven private hospitals in Cape Town from 1 January 2011 to 31 October 2016. Meteorological data were obtained from the South African Weather Service (SAWS). A quasi-Poisson regression model was used to investigate the association between TV and health outcomes after controlling for potential effect modifiers. A positive and statistically significant association between TV and hospital admissions for both diseases was observed, even after controlling for the non-linear and delayed effects of daily mean temperature and relative humidity. TV showed the greatest effect on the entire study group when using short lags, 0–2 days for CVD and 0–1 days for RD hospitalisations. However, the elderly were more sensitive to RD hospitalisation and the 15–64 year age group was more sensitive to CVD hospitalisations. Men were more susceptible to hospitalisation than females. The results indicate that more attention should be paid to the effects of temperature variability and change on human health. Furthermore, different weather and climate metrics, such as TV, should be considered in understanding the climate component of the epidemiology of these (and other diseases), especially in light of climate change, where a wider range and extreme climate events are expected to occur in future.

## 1. Introduction

Non-communicable diseases (NCDs) such as cardiovascular disease (CVD) and respiratory diseases (RD) are among the top causes of mortality and morbidity globally [1,2]. According to the World Health Organization (WHO), nearly 86% of NCD deaths occur in low- and middle-income countries (LMICs) [3]. Although the occurrence of NCDs in high-income countries declined in the past decades, there is evidence that the incidence and prevalence of CVD and RD mortality and morbidity have increased in LMICs [3]. Particularly, between 2016 and 2018, in South Africa, mortality attributable to CVD (I00–I99) increased from 18.6 percent to 18.9 percent. In contrast, mortality attributable to RD (J00–J99) decreased from 9.4 percent to 9.1 percent [4,5]. Knowledge of the incidence and prevalence of NCDs in Africa is poor, and people lack an understanding of the risk factors and clinical symptoms associated with CVD or RD health outcomes [4,5]. These risk factors include weather and meteorological variables such as ambient mean temperature and temperature variability (TV) [6,7,8,9].

It is known that weather events and climate trends have an impact on human health. Extreme weather events, which are anticipated to intensify with climate change (including significant temperature extreme events), pose a grave danger to human health [10,11,12]. Future climate projections indicate that global temperatures are likely to continue to rise throughout the 21st century. It is projected that, on average, annual increases of 1–3 °C in global temperatures may occur by 2050 [13], which will be amplified in southern Africa [10]. By 2100, warming is projected to increase the average temperature by 3–4 °C along the South African coast and by 6–7 °C inland, surpassing the anticipated global warming averages [14]. Several epidemiological studies attributed non-optimal temperature extremes, including heatwaves, extreme cold events, and extreme temperatures, to increased hospital admissions and mortality from non-communicable and infectious diseases [6,8,15,16,17,18]. Few studies have investigated the relationship between ambient temperature and health outcomes in Africa [7], and only one published paper, which considered temperature variation in Africa could be located [18]. Temperature variability (TV) is an important meteorological indicator reflecting climate changes, such as rapid intra- and inter-day temperature changes [8,19,20].

Globally, evidence on the short-term CVD and RD effects of TV is increasing [5,8,19,20,21,22,23,24]. The majority of these studies reported TV to be associated with increased risks of CVD and RD health outcomes. However, there are still some inconsistencies in the association between TV and hospital admissions [22]. For example, one study conducted in Bangladesh found no association between TV and RD emergency hospital admissions [22], while a Korean study found that temperature change was associated with increased hospital admissions for total respiratory diseases [25]. Some of the limitations of these studies include the selection of study participants, a cohort of elderly (older than 65) volunteers [26], and focus on a group older than 35 years of age [24]; by focusing on one age group, the effects of TV on the general population might be over- or underestimated. The majority of these studies focused on the health effects of intra-day (e.g., diurnal temperature) [18,27,28,29] and inter-day (e.g., temperature change between neighbouring days and the standard deviation of daily mean summer temperature) [30,31]. The associations between temperature variability (TV) and population health may be better explained by a composite index accounting for the effects of intra-day and inter-day variability since the impact of TV can last for several days after exposure [8,19,21]. Few studies assessed the detrimental effects of temperature changes using the composite index of TV on cardiovascular and respiratory disease hospital admissions [19,22,32]. There are even fewer studies that comparatively assessed TV’s effects on cardiovascular and respiratory disease hospital admissions [22], especially in developing countries such as South Africa.

This study applied the time-series epidemiological study design to evaluate the association between short-term TV and CVD and RD hospitalisation in the City of Cape Town, South Africa, between 1 January 2011 and 31 October 2016. Vulnerability by different age groups (0–14, 15–64, and ≤65 years) and sex was assessed. Delayed effects of temperature on TV and health outcome were investigated using distributed lag non-linear models (DLNM) framework [33].

## 2. Materials and Methods

### 2.1. Study Location

**Figure 1:**
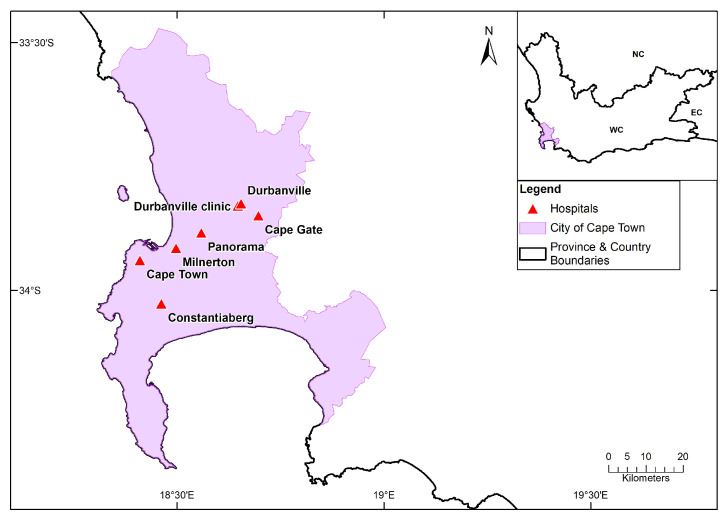
The location of the private hospitals in the City of Cape Town region that were considered in the study.

A list of potential private hospitals that were included in this study was obtained from the Hospital Association of South Africa (HASA) website (https://hasa.co.za/, accessed on 30 June 2019). Only seven private hospitals located in the City of Cape Town, South Africa, were included in this study due to the availability of data (Figure 1).

### 2.2. Data Collection

Daily counts of hospital admissions at the seven private hospitals in the City of Cape Town from 1 January 2011 to 31 October 2016 were data supplied by the respective hospital authorities. Hospitalisations were classified on the primary diagnosis and according to the International Classification of Diseases 10th Revision codes: RD (J00–J99) and CVD (I00–I99). The hospital admission information also included age groups (all ages combined, 0–14 years, 15–64 years, and ≥65 years) and sex.

Daily temperature (degrees Celsius), relative humidity (%), wind speed (km/h), rainfall (mm), and barometric pressure (kPa) data for the Cape Town-Worcester weather station were obtained from the South African Weather Service (SAWS). The ethical approval reference number is 738/2019.

### 2.3. Exposure Definition

TV was calculated as a composite of intra-day and inter-day variability using the minimum and maximum temperatures [17]. For example, TV for the preceding 3 days’ exposure was calculated as follows: *TV*${}_{0-3}$ = standard deviation of the minimum temperature at lag 0, the maximum temperature at lag 0, the minimum temperature at lag 1, maximum temperature lag at 1, the minimum temperature at lag 2, and maximum temperature at lag 2. The general equation to calculate TV is shown below:

(1) $$TV_{0-i}=\sqrt{\frac{\sum_{i=1}^{n}{\left(T_{i}-\bar{T}\right)}^{2}}{n-1}}$$

where $T_{i}$ is either maximum or minimum temperatures during exposure days, *n* is the number of observations, and $\bar{T}$ is the average of the minimum and maximum temperatures during exposure days expressed as follows:

(2) $${\bar{T}}_{1}=\sum_{i=1}^{n}\frac{T_{i}}{n_{i}}$$

### 2.4. Statistical Analysis

The association between TV and hospital admissions was investigated using a generalised linear regression model and assuming a quasi-Poisson distribution, allowing for an over-dispensed hospital admission count [34]. The relationship between exposure to TV and hospital admissions was first explored by employing a natural cubic spline with different degrees of freedom and by examining various TV options as exposure variables. Furthermore, the analysis of variance (ANOVA) test and the value of quasi-Akaike information criterion (QAIC) confirmed that the models with a cubic spline better capture the effects of TV on hospital admissions, which is in line with previous studies [17,29,35].

QAIC goodness of fit for over-dispersed count data was used to select the best model options. Long-term trends and seasonality were controlled using a natural cubic spline with 7 degrees of freedom per year. Categorical variables were used to control for confounding effects of the day of the week and public holidays. Relative humidity was controlled for as a natural cubic spline with 3 degrees of freedom.

The mean temperature was added to the models as a distributed nonlinear lag function accounting for both nonlinear and delayed effects. Several options for the cross-basis of the distributed nonlinear lag function were explored, and the cross-basis with the lowest QAIC was selected. A natural cubic spline with 4 degrees of freedom was used both for the daily mean temperature and the lags (0 to 21 days). Three internal knots were placed at equally spaced percentiles (25th, 50th, and 75th) and two internal knots were placed at equally spaced log-values of lag (at 1.42 and 5.45 days) plus intercept. This is a similar approach to those in previous studies [8,15]. The regression model used can be expressed as follows:

(3) $$E(\log\left(Y_{i})\right)=\alpha +\beta TV_{0-i}+\lambda dow_{i}+\sigma pub_{i}+ns\left(RH,3\right)+ns\left(time_{i},7\times 6\right)+cb.tmean$$

where $Y_{i}$ is the outcome variable on day *i*, *TV*${}_{0-i}$ represents temperature variability on day *i* with 0 to 7 days of lag, dow${}_{i}$ and pub${}_{i}$ are categorical variables controlling for day of week and public holiday variability, ns(time${}_{i}$, 7 × 6) is the natural splines of calendar time, and cb.tmean is the crossbasis function for daily mean temperature. The associations along with 95% confidence intervals are reported as percent change in CVD or RD hospitalisations per interquartile range increase in TV.

### 2.5. Sensitivity Analysis

To test for the robustness of the results, sensitivity analyses were performed firstly by changing the degrees of freedom in time per year (df = 3–8), the natural spline for temperature (df = 3–6), and the spline of relative humidity (df = 3–6). Secondly, the maximum lag for the cross-basis function of temperature was changed from 21 to 28 to examine whether using 21 lag days was sufficient to control for the temperature effects on health outcomes [8]. Thirdly, relative humidity was explored as different options, including a categorical variable, a cubic spline with varying degrees of freedom. The models were also adjusted for air pollutants, namely, nitrogen dioxide (NO${}_{2}$), sulphur dioxide (SO${}_{2}$), and particulate matter with a diameter of 10 microns or less (PM${}_{10}$). Furthermore, stratified analyses by age groups and sex were also conducted to identify the susceptible population and seasonal variation of the TV–hospital admission association. All statistical analyses in this study were conducted using the R Statistical Software (v4.1.2; R Core Team 2022), where the “splines” and “DLNM” software packages were used to fit the relationship between TV and schizophrenic hospitalisations. *p* values of ≤0.05 (two-sided) were considered for statistical significance.

## 3. Results

Table 1 presents the demographic characteristics of the hospitalisations at the seven private hospitals in the City of Cape Town from 1 January 2011 to 31 October 2016. A total of 58,818 CVD and 54,317 RD hospitalisations were recorded during the study period. Most RD hospitalisations were for children aged between 0 and 14 years (49%), whereas the same age group had the least CVD hospitalisations. The 0–14 year age group was excluded from the CVD hospitalisation subgroup analysis. The majority (49.9%) of CVD hospitalisations were among the elderly (≤65 years). The distribution of hospitalisations by sex was similar for both diseases.

**Figure 2:**
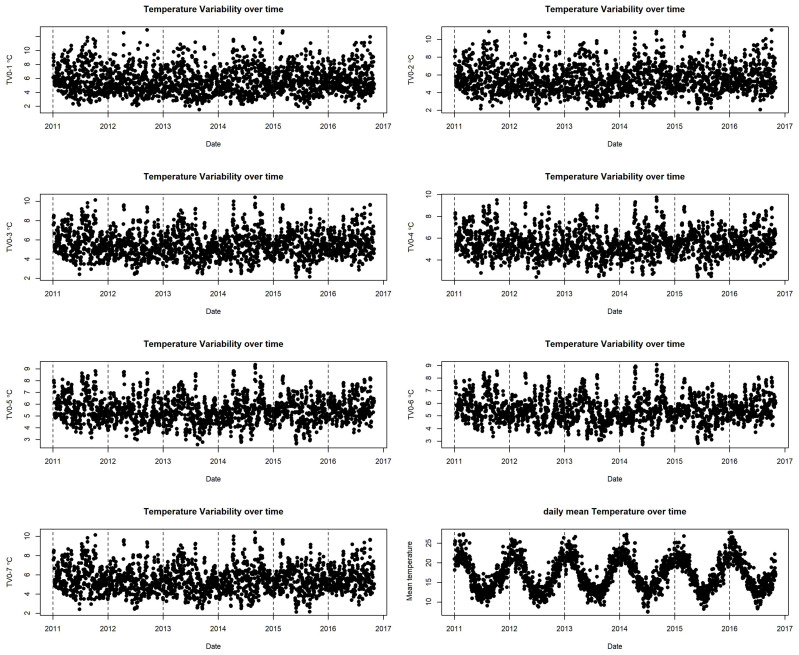
Time series of temperature and temperature variability over different exposure days.

Figure 2 illustrates the time series of TV at different exposure days (0–1 days to 0–7 days) and a time series of daily mean temperature. The TV distribution at different exposure days is similar to that in Table 2. The annual-average daily mean temperature was 17.1 °C, with a range of 7.52 °C to 27.8 °C. The annual-average TV for the preceding 2 days’ exposure (TV${}_{0-1}$) was 5.70, with a range of 1.52 °C to 13.0 °C.

Table 3 shows the percent change in CVD and RD hospitalisations associated with an interquartile range increase in TV for the entire study group. In general, positive and statistically significant associations between TV and hospitalisations for both diseases were observed. The effect of TV on hospitalisations was immediate for both diseases. Per Inter quartile increase (IQR), the highest increase in CVD hospitalisations, 6.04% (95% CI: 3.15–9.01%), was observed at 0–3 days of exposure; after that, the risk started to decrease until 0–7 days of exposure. The effect of TV on RD hospitalisations reached a peak after 2 days.

After adjusting for the day of the week, time, and seasonal trends and the effect of public holidays (Table 4), the effect of TV on both CVD and RD hospitalisations remained statistically significant. However, the magnitude of the risks decreased. For the entire study group, the highest increase in CVD hospitalisations (2.84%, 95% CI: 1.44–4.27%) was observed at 0–2 exposure days and, at 0–1 days for RD hospitalisations (2.79%, 95% CI: 1.44–4.17%) thereafter, started to decrease gradually.

Table 4 shows the associations between TV and hospitalisations for different age groups. The effect estimates varied by exposure days for the different age groups; for example, when 0–1 day of exposure was considered, the highest risk for hospitalisation was observed in the elderly for both diseases. When 0–5 days of exposure were investigated, the highest increase in CVD hospitalisations, 3.01% (95% CI: 1.17–4.89%), was observed for the 15–64 year age group, whereas the highest effect estimates, 4.74% (95% CI: 2.02–7.50%), were observed for the 65 years or older age group.

Susceptibility differed by gender (Table 4). In general, males were more vulnerable to CVD hospitalisation due to exposure to TV than females. The highest increase in CVD hospitalisation (3.42%, 95% CI: 1.68–5.18%) for males was observed at 0–5 days of exposure, whereas the risk of CVD hospitalisation (2.76%, 95% CI: 0.70%, 4.86%) for females reached a maximum after 7 days of exposure. For RD hospitalisation, the highest increases in hospitalisations for both females (2.65%, 95% CI: 0.91–4.43%) and males (2.90%, 95% CI: 1.14–4.69%) appeared at 0–1 days of exposure. However, the males were more at risk compared to females. The risk for RD hospitalisation for both females and males remained stable but started to decrease after 0–2 days of exposure.

**Figure 3:**
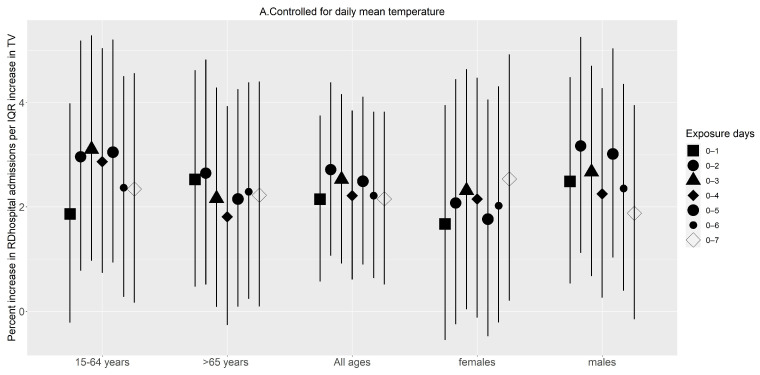

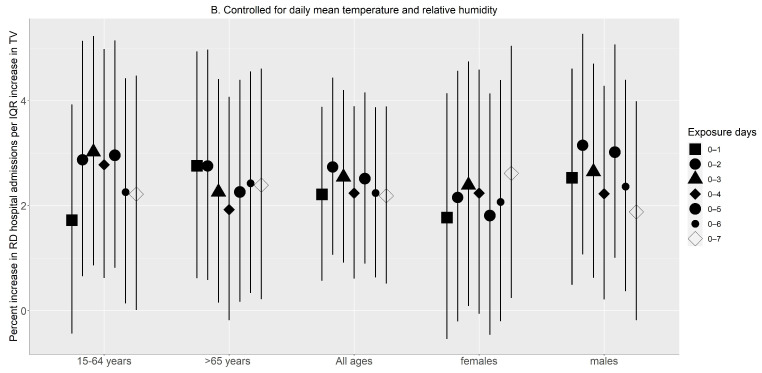
Percent change (95% confidence interval) in cardiovascular disease hospitalisation associated with an interquartile increase in temperature variability (°C) on different exposure days: (**A**) After controlling for the day of the week, time, and seasonal trends and daily mean temperature. (**B**) After controlling for the day of the week, time, and seasonal trends and daily mean temperature and relative humidity.

**Figure 4:**
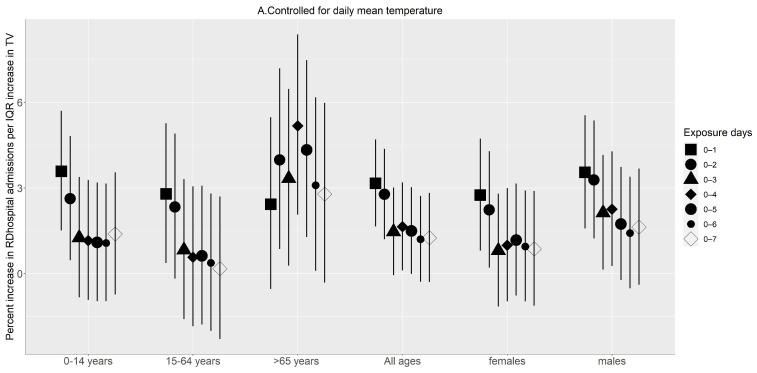

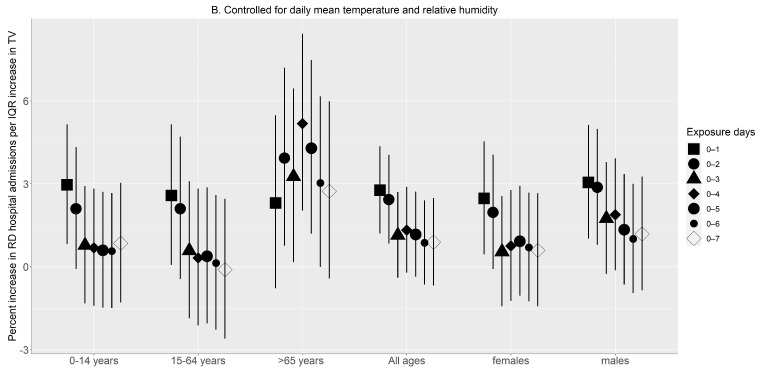
Percent change (95% confidence interval) in respiratory disease hospitalisation associated with an interquartile increase in temperature variability (°C) on different exposure days: (**A**) After controlling for day of the week, time, and seasonal trends and daily mean temperature. (**B**) After controlling for the day of the week, time, and seasonal trends and daily mean temperature and relative humidity.

Further controlling the models for the effects of daily mean temperature, the highest effect estimates for the entire study group appeared at different exposure days. The highest effect estimates appeared at 0–2 days for CVD hospitalisation (Figure 3A) and at 0–1 day for RD hospitalisation (Figure 4A). After reaching the maximum, the effect estimates of TV on hospitalisations for both diseases tended to be stable and then decreased. The subgroup analysis showed that the 15–64 age group was more vulnerable to CVD hospitalisations and the 65 or older age group was more vulnerable to RD hospitalisations. Similar patterns Figure 4A,B were observed after controlling for all other covariates, including relative humidity.

For the two genders (Table A1), per IQR increase, the greatest effect of TV on CVD hospital admissions occurred in TV at 0–2 days of exposure for males (3.15%, 95% CI: 1.07–5.27%) and at 0–7 days of exposure for females (2.62%, 95% CI: 0.24–5.04%). The effect of TV on the number of hospital admissions for RD reached a peak at 0–1 days of exposure for both males (2.47%, 95% CI: 0.45–4.53%) and females (3.05%, 95% CI: 1.02–5.13%). After reaching the maximum, the effect estimates tended to remain stable and then decreased.

The analysis was repeated for the main results using different model options for all health outcomes and subgroups but only reported the results for the entire study group in Table A2, Table A3 and Table A4. For both health outcomes, the sensitivity analysis showed that the associations were robust to changes in degrees of freedom for time, spline of temperature, and natural cubic spline of relative humidity. The results remained unchanged when the maximum number of lags for the crossbasis of daily mean temperature changed. Using daily minimum temperature instead of daily mean temperature did not change the results. However, when maximum temperature was used, the results attenuated. Furthermore, adding relative humidity as a linear term or as a categorical variable to the model did not change the results.

The main results were repeated using different model options for all health outcomes and subgroups but only reported the results for the entire study group in Table A2, Table A3 and Table A4. For both health outcomes, the sensitivity analysis showed that the associations were robust to changes in degrees of freedom for time, spline of temperature, and natural cubic spline of relative humidity. The results remained unchanged when the maximum number of lags for the daily mean temperature cross basis changed. Using daily minimum temperature instead of daily mean temperature did not change the results. However, when maximum temperature was used, the effects attenuated. Furthermore, adding relative humidity as a linear term or as a categorical variable to the model did not change the results. However, previous studies ascertained the robustness and independence of temperature-related health studies with or without the influence of air pollution [9,36]. In the current study, the effect estimates did not change after controlling for the effects of PM${}_{10}$ and SO${}_{2}$. However, the magnitude of the effect estimates decreased after controlling for NO${}_{2}$.

## 4. Discussion

This is the first local epidemiological study in Southern Africa to evaluate evidence documenting the cardio-respiratory health effects of TV. In general, positive and statistically significant impacts of TV exposure were observed. The 15–64 age group was more vulnerable to CVD hospitalisation and the elderly (65 years or older), were more vulnerable to RD hospitalisation due to TV exposure. Men appeared to be more susceptible to hospitalisation than females.

Few studies evaluated TV’s effects on CVD and RD health outcomes [19,22,23,32,37]. These studies found a more significant effect of TV on RD health outcomes than CVD health outcomes. Contrary to these studies, in this study, the effect of TV was higher on CVD hospitalisations on most exposure days, except at shorter exposure days (0–1 and 0–2 days), where the effect of TV on RD hospitalisations was higher for all subgroups. The results might be different due to different health outcomes and geographic locations. In this study, the effects of TV on hospital admissions, instead of mortality, were explored.

For the elderly, the most significant effect of TV on CVD hospitalisations occurred at short TV exposure (0–1 days). For the entire study group and males, the most significant effect of TV on CVD hospitalisations occurred at 0–2 days of exposure. For the 15–64 age group, the most significant effect of TV on CVD hospitalisations occurred at 0–3 days of exposure. For females, the most significant effect of TV on CVD hospitalisations occurred at 0–7 days of exposure. This indicates that TV had acute effects on the entire study group, males, and the elderly for the incidence of CVD hospitalisations. In contrast, the effect of TV on the incidence of CVD hospitalisations for the 15–64 age group and females was delayed. Tian et al. (2019) [19] also observed the acute effects of TV on CVD hospitalisations for the entire study group. In contrast, Luo et al. (2017) [38] and Zhang et al. (2017) [29] reported the strongest effects of TV on CVD mortality at longer exposures.

TV had acute effects on the incidence of RD hospitalisations for the entire study group, all genders, and the 0–14 year and 15–64 year old age groups occurring at 0–1 days of exposure, except for the elderly, where the greatest effect of TV on RD hospitalisations occurred at 0–4 days of exposure. This is in line with the results from a previous study on the effects of TV on the common cold (ICD code: J00) using the diurnal temperature range as an indicator for TV. They observed the highest effects at lag 0 for the entire study group and the ≤15 and 15–65 year age groups. While for the older than 65 year age group, the highest effects were detected at lag 5 [28]. A recent study conducted in Bangladesh also observed delayed effects of TV on RD hospitalisations in the elderly group, with the highest effects observed at 0–7 days of exposure [22]. These results further highlight the importance of considering different exposure days and lags when assessing the health burden of TV. Thus, the composite index of TV accounting for continuous intra- and inter-day temperatures may be a better indicator than intra-day temperatures alone [23].

For CVD hospitalisations, the highest estimate of the effects of TV between the two age groups fluctuated for different exposure days. The 15–64 age groups were more sensitive to TV effects on most exposure (0–2 days and 0–6 days) compared to the elderly. The use of air conditioning may be one of the potential reasons why young adults are generally more sensitive to temperature changes since young people in urban areas such as in the City of Cape Town tend to spend more time indoors or in office spaces. Air conditioners (AC) help to regulate indoor temperatures; some researchers argue that the use of AC make people physically and mentally dependent and acclimatised to stable temperatures, which makes them susceptible to temperature variability [23,39]. These results are consistent with the results observed by Tian et al. (2019) [19], where young people in the 18–64 age group (0.81%, 95% CI: 0.59–1.03%) were more at risk of CVD hospitalisations due to TV exposure as compared with the 65–74 age group (0.19%, 95% CI: 0.03–0.34%) and the older than 75 age group (0.55%, 95% CI: 0.34–0.75%).

Numerous studies have reported that the effects of temperature change vary by age group, with the elderly being more sensitive [22,23,28,32]. Similar to previous studies, the current study also found that the elderly were more susceptible to RD hospitalisations after exposure to TV than all other age groups. Older people may be prone to the grave effects of TV due to declining thermoregulatory function and poor acclimatisation skills [19]. Previous studies also demonstrated that temperature variation is associated with heart rate, blood cholesterol levels, blood pressure, peripheral vasoconstriction, platelet viscosity, plasma fibrinogen concentrations, and the immune system’s ability to resist infectious agents [28,40]. These physiological changes may trigger cardiovascular and respiratory diseases [19].

Several studies stated that gender matters when exploring weather-related effects on health [6,27,38]. Similar to previous studies that assessed the effects of temperature change [31], diurnal temperature range, and temperature change between neighbouring days [20], we also observed that, between the two genders, men were generally more sensitive to the effects of TV on CVD hospitalisations than females, except at 0–7 days of exposure, where females appeared to be more sensitive than males. A Brazil study [32] also observed that females were affected by prolonged exposure to TV. Furthermore, men were more sensitive to the effects of TV on RD hospitalisations on all exposure days. Men may appear to be more vulnerable to TV effects due to spending longer time outdoors for activities, thereby incurring a greater risk of exposure to TV. In contrast, Chinese [38] and Brazilian [32] studies observed stronger effects of TV on females. Such a discrepancy may be due to methodological and socioeconomic differences.

Not controlling for the effects of daily mean temperature in TV and health outcome associations could overestimate the health risks associated with TV, as shown in Figure 3, Figure 4, and Table A1. Previous studies showed that daily mean temperature could confound the association when assessing the effects of temperature variability on health outcomes [8]. Similarly, in this study, TV was associated with increased risks of hospital admission even after controlling for the main effects of daily mean temperature and relative humidity. This ascertains that TV is a health risk factor in the City of Cape Town and is independent of daily mean temperatures. Similar to the studies by Cheng et al. (2017) [41] and Guo et al. (2016) [8], controlling for the effects of daily mean temperature decreased the magnitude of the effect estimates and became non-significant in some instances.

This study has some strengths. Firstly, this is the first study in Southern Africa to evaluate the effects of TV on hospital admissions. Secondly, the study investigated the effects of TV on cause-specific hospital admissions rather than all-cause/non-accidental health effects, as in several other studies. Thirdly, we conducted subgroup analysis by age groups (0–14, 15–64 and ≤65-year-old) and gender (females and males) to evaluate sensitivity by different subpopulations. Fourthly, the nonlinear and delayed effects of daily mean temperature were assessed using flexible distributed lag nonlinear models (DLNM). Fifthly, the interactive effects of temperature variability and air pollution were investigated. Lastly, a range of sensitivity analyses was performed to evaluate the robustness of our results.

This study also has some limitations. Firstly, like other time series studies, individual exposure data were not used to assess the effects of TV. Exposure data from several fixed stations were used instead. This is known to create measurement errors in an exposure. These measurement errors are likely to be random, typically resulting in an underestimation of exposure-related risks [8,42]. Secondly, we only collected data on the number of RD and CVD hospitalisations from seven of the many private hospitals in the City of Cape Town, which may not be enough to extrapolate the results to represent the entire population in South Africa fully [9]. Finally, there are many factors that contribute to early onset of respiratory and cardiovascular diseases such as social economic status, diet, smoking habits, alcohol, and weight, these factors could not be accounted for in this study since the study was conducted at the population level. More studies are warranted to expand on the interactive effects of TV and air pollutants on health outcomes.

## 5. Conclusions

In conclusion, this study, which to, our knowledge is the first study in South Africa, demonstrated that daily temperature variation is associated with increased risks of CVD and RD hospital admissions in the City of Cape Town. The results add to current studies that aims to understand health implications of climate change and to provide scientific guidelines to assist local government in CVD and RD control and prevention in the country. These findings may have implications for assessing health risks associated with meteorological conditions and for developing group-specific adaptation strategies to reduce the grave effects of climate change. 

## Figures and Tables

**Table 1:** Summary statistics of respiratory and cardiovascular diseases hospital admissions in the City of Cape Town by age and sex, from 1 January 2011 to 31 October 2016.

Variable	Cardiovascular Diseases	Respiratory Diseases
**Age (Years)**		
Total	54,818	58,317
0–14 (%)	498 (0.908)	28,518 (48.9)
15–64 (%)	27,225 (49.7)	19,418 (33.3)
≥65	27,095 (49.9)	10,381 (17.8)
**Gender**		
females	22,914 (41.8)	29,741 (51)
males	31,904 (58.2)	28,576 (49)

**Table 2:** Distribution of weather conditions and temperature variability at different exposure days in the City of Cape Town, 1 January 2011–31 October 2016.

Variable	Mean	Min	P25	Median	P75	Max
Tmean (°C)	17.1	7.52	13.8	16.8	20.2	27.8
RH (%)	70.9	35.7	64.4	71.1	77.8	99.1
**Temperature variability**						
$TV_{0-1}$ (°C)	5.70	1.52	4.32	5.41	6.86	13.0
$TV_{0-2}$ (°C)	5.55	2.08	4.43	5.35	6.52	11.1
$TV_{0-3}$ (°C)	5.51	2.14	4.57	5.40	6.33	10.4
$TV_{0-4}$ (°C)	5.50	2.43	4.66	5.39	6.23	9.76
$TV_{0-5}$ (°C)	5.50	2.57	4.73	5.38	6.14	9.36
$TV_{0-6}$ (°C)	5.50	2.73	4.81	5.40	6.09	9.06
$TV_{0-7}$ (°C)	5.50	2.84	4.86	5.40	6.09	9.03

**Table 3:** Percent change (mean and 95% CI) of CVD and RD hospitalisations associated with an interquartile range (IQR) increase in temperature variability (°C) on different exposure days for all ages combined; models were not adjusted for any covariates.

	Percentage Increase in Hospitalisations (%)	
Exposure Days	Cardiovascular Diseases	Respiratory Diseases
0–1	3.90 (0.96, 6.93)	4.17 (1.38, 7.03)
0–2	5.97 (2.99, 9.04)	4.68 (1.88, 7.56)
0–3	6.04 (3.15, 9.01)	4.55 (1.85, 7.33)
0–4	4.83 (1.97, 7.77)	4.45 (1.75, 7.21)
0–5	3.96 (1.18, 6.83)	3.94 (1.31, 6.64)
0–6	2.96 (0.26, 5.73)	3.41 (0.86, 6.04)
0–7	2.48 (−0.28, 5.32)	3.19 (0.57, 5.87)

**Table 4:** Percent change (mean and 95% CI) of CVD and RD hospitalisations associated with an interquartile range (IQR) increase in temperature variability (°C) on different exposure days, adjusting for time trends and seasonal variation, day of the week, and public holidays.

	Percent Increase in Cardiovascular Disease Hospitalisations (%)						
	Exposure Days						
**Group**	**0–1**	**0–2**	**0–3**	**0–4**	**0–5**	**0–6**	**0–7**
All	1.91 (0.50, 3.35)	2.60 (1.15, 4.06)	2.68 (1.27, 4.11)	2.56 (1.15, 3.99)	2.84 (1.44, 4.27)	2.63 (1.23, 4.04)	2.61 (1.15, 4.08)
15–64	1.72 (−0.14, 3.61)	2.75 (0.85, 4.69)	2.97 (1.11, 4.85)	2.84 (0.98, 4.73)	3.01 (1.17, 4.89)	2.46 (0.63, 4.32)	2.37 (0.47, 4.32)
≥65	2.17 (0.34, 4.04)	2.59 (0.73, 4.48)	2.56 (0.74, 4.40)	2.48 (0.66, 4.34)	2.86 (1.04, 4.70)	3.00 (1.19, 4.84)	3.07 (1.18, 4.99)
Females	1.21 (−0.77, 3.23)	1.74 (−0.28, 3.80)	2.16 (0.18, 4.17)	2.20 (0.21, 4.22)	2.05 (0.08, 4.06)	2.31 (0.35, 4.31)	2.76 (0.70, 4.86)
Males	2.43 (0.68, 4.20)	3.22 (1.44, 5.03)	3.05 (1.32, 4.81)	2.82 (1.08, 4.58)	3.42 (1.68, 5.18)	2.85 (1.14, 4.60)	2.50 (0.71, 4.32)
	**Percent Increase in Respiratory Disease Hospitalisations (%)**						
**Group**	**0–1**	**0–2**	**0–3**	**0–4**	**0–5**	**0–6**	**0–7**
All	2.79 (1.44, 4.17)	2.53 (1.15, 3.92)	1.61 (0.28, 2.96)	1.83 (0.50, 3.19)	1.78 (0.45, 3.12)	1.58 (0.27, 2.91)	1.66 (0.29, 3.04)
0–14	2.98 (1.12, 4.87)	2.26 (0.38, 4.18)	1.34 (−0.48, 3.20)	1.39 (−0.43, 3.25)	1.47 (−0.33, 3.32)	1.52 (−0.28, 3.35)	1.83 (−0.04, 3.73)
15–64	1.85 (−0.31, 4.04)	1.48 (−0.69, 3.71)	0.50 (−1.60, 2.64)	0.46 (−1.64, 2.62)	0.61 (−1.48, 2.76)	0.53 (−1.55, 2.65)	0.43 (−1.74, 2.64)
≥ 65	3.74 (1.05, 6.50)	5.06 (2.31, 7.89)	4.41 (1.73, 7.15)	5.65 (2.93, 8.45)	4.74 (2.05, 7.50)	3.59 (0.95, 6.30)	3.24 (0.50, 6.06)
Females	2.65 (0.91, 4.43)	2.27 (0.50, 4.07)	1.23 (−0.48, 2.96)	1.40 (−0.31, 3.15)	1.50 (−0.21, 3.24)	1.31 (−0.38, 3.03)	1.25 (−0.51, 3.05)
Males	2.90 (1.14, 4.69)	2.73 (0.95, 4.55)	2.00 (0.26, 3.76)	2.24 (0.50, 4.00)	1.99 (0.27, 3.74)	1.82 (0.12, 3.56)	2.05 (0.27, 3.86)

## Data Availability

Meteorological data can be requested from the South African Weather Service https://www.weathersa.co.za/ (accessed on 30 June 2019).

## Appendix A

**Table A1:** Percent change (mean and 95% CI) of CVD and RD hospitalisations associated with an interquartile range (IQR) increase in temperature variability (°C) on different exposure days, adjusting for time trends and seasonal variation, day of the week, public holidays, and daily mean temperature.

	Percent Increase in Cardiovascular Disease Hospitalisations (%)						
	Exposure Days						
**Group**	**0–1**	**0–2**	**0–3**	**0–4**	**0–5**	**0–6**	**0–7**
All	2.15 (0.57, 3.75)	2.71 (1.07, 4.39)	2.53 (0.92, 4.16)	2.22 (0.61, 3.85)	2.49 (0.90, 4.11)	2.22 (0.63, 3.82)	2.16 (0.51, 3.83)
15–64	1.86 (−0.21, 3.99)	2.96 (0.78, 5.19)	3.10 (0.97, 5.29)	2.87 (0.74, 5.04)	3.05 (0.93, 5.21)	2.37 (0.28, 4.50)	2.34 (0.17, 4.56)
≤65	2.52 (0.47, 4.62)	2.65 (0.51, 4.82)	2.16 (0.08, 4.28)	1.81 (−0.26, 3.93)	2.15 (0.09, 4.26)	2.29 (0.24, 4.39)	2.22 (0.09, 4.40)
Females	1.68 (−0.55, 3.95)	2.07 (−0.25, 4.45)	2.31 (0.04, 4.64)	2.15 (−0.12, 4.47)	1.77 (−0.48, 4.06)	2.02 (−0.21, 4.31)	2.54 (0.20, 4.92)
Males	2.49 (0.53, 4.49)	3.17 (1.12, 5.25)	2.67 (0.68, 4.70)	2.25 (0.26, 4.28)	3.01 (1.03, 5.03)	2.36 (0.39, 4.36)	1.88 (−0.15, 3.95)
	**Percent increase in respiratory disease hospitalisations (%)**						
**Group**	**0–1**	**0–2**	**0–3**	**0–4**	**0–5**	**0–6**	**0–7**
All	3.17 (1.66, 4.70)	2.78 (1.21, 4.37)	1.47 (−0.05, 3.02)	1.65 (0.12, 3.20)	1.50 (−0.01, 3.03)	1.21 (−0.28, 2.72)	1.26 (−0.29, 2.83)
0–14	3.59 (1.51, 5.70)	2.63 (0.47, 4.83)	1.26 (−0.83, 3.39)	1.16 (−0.92, 3.28)	1.10 (−0.96, 3.19)	1.08 (−0.96, 3.15)	1.39 (−0.73, 3.55)
15–64	2.79 (0.37, 5.27)	2.33 (−0.17, 4.90)	0.83 (−1.59, 3.31)	0.58 (−1.84, 3.06)	0.63 (−1.78, 3.08)	0.38 (−2.00, 2.81)	0.18 (−2.29, 2.70)
≤ 65	2.43 (−0.53, 5.48)	3.98 (0.87, 7.19)	3.33 (0.29, 6.47)	5.18 (2.07, 8.38)	4.34 (1.28, 7.48)	3.10 (0.11, 6.18)	2.79 (−0.31, 5.99)
Females	2.75 (0.81, 4.73)	2.23 (0.21, 4.29)	0.81 (−1.15, 2.80)	1.00 (−0.96, 3.00)	1.17 (−0.77, 3.16)	0.95 (−0.97, 2.91)	0.87 (−1.12, 2.90)
Males	3.55 (1.58, 5.55)	3.28 (1.24, 5.37)	2.13 (0.14, 4.16)	2.26 (0.27, 4.29)	1.74 (−0.22, 3.74)	1.42 (−0.51, 3.40)	1.63 (−0.38, 3.68)

**Table A2:** Results of sensitivity analyses by changing the degrees of freedom for time per year on the association between TV, and cardiovascular and respiratory disease hospital admissions on all exposure days for all ages. The results were scaled per IQR increase in TV.

	Percent Increase in Cardiovascular Disease Hospitalisations (%)						
	Exposure Days						
**Degrees of Freedom**	**0–1**	**0–2**	**0–3**	**0–4**	**0–5**	**0–6**	**0–7**
3	2.32 (0.68, 3.98)	2.92 (1.27, 4.60)	2.74 (1.15, 4.36)	2.45 (0.88, 4.05)	2.63 (1.07, 4.21)	2.35 (0.81, 3.91)	2.31(0.73, 3.92)
4	2.19 (0.56, 3.85)	2.75 (1.10, 4.42)	2.57 (0.98, 4.18)	2.29 (0.71, 3.89)	2.48 (0.93, 4.06)	2.22 (0.68, 3.78)	2.22 (0.63, 3.83)
5	2.16 (0.51, 3.83)	2.79 (1.12, 4.49)	2.66 (1.04, 4.30)	2.39 (0.78, 4.02)	2.66 (1.06, 4.28)	2.44 (0.85, 4.04)	2.45 (0.81, 4.11)
6	2.23 (0.59, 3.90)	2.80 (1.13, 4.49)	2.64 (1.03, 4.28)	2.35 (0.74, 3.98)	2.61 (1.01, 4.24)	2.35 (0.76, 3.96)	2.31 (0.66, 3.99)
7	2.21 (0.57, 3.88)	2.74 (1.06, 4.44)	2.55 (0.92, 4.20)	2.24 (0.61, 3.89)	2.51 (0.90, 4.16)	2.24 (0.64, 3.87)	2.19 (0.52, 3.89)
8	2.23 (0.59, 3.90)	2.80 (1.13, 4.49)	2.64 (1.03, 4.28)	2.35 (0.74, 3.98)	2.61 (1.01, 4.24)	2.35 (0.76, 3.96)	2.31 (0.66, 3.99)
	**Percent increase in respiratory disease hospitalisations (%)**						
**Degrees of freedom**	**0–1**	**0–2**	**0–3**	**0–4**	**0–5**	**0–6**	**0–7**
3	3.73 (2.14, 5.35)	3.48 (1.87, 5.11)	2.31 (0.77, 3.87)	2.56 (1.03, 4.11)	2.37 (0.86, 3.90)	2.10 (0.62, 3.60)	2.21 (0.68, 3.76)
4	3.45 (1.87, 5.05)	3.14 (1.55, 4.76)	1.93 (0.40, 3.48)	2.13 (0.61, 3.67)	1.92 (0.42, 3.43)	1.65 (0.19, 3.14)	1.75 (0.23, 3.29)
5	3.03 (1.46, 4.62)	2.71 (1.12, 4.32)	1.51 (−0.02, 3.06)	1.72 (0.20, 3.27)	1.53 (0.02, 3.06)	1.26 (−0.22, 2.77)	1.31 (−0.23, 2.87)
6	2.74 (1.18, 4.32)	2.45 (0.87, 4.05)	1.22 (−0.30, 2.76)	1.44 (−0.07, 2.98)	1.29 (−0.21, 2.82)	1.03 (−0.46, 2.53)	1.08 (−0.47, 2.64)
7	2.77 (1.21, 4.36)	2.43 (0.84, 4.05)	1.14 (−0.40, 2.70)	1.33 (−0.21, 2.90)	1.17 (−0.36, 2.72)	0.87 (−0.64, 2.40)	0.89 (−0.68, 2.49)
8	2.74 (1.18, 4.32)	2.45 (0.87, 4.05)	1.22 (−0.30, 2.76)	1.44 (−0.07, 2.98)	1.29 (−0.21, 2.82)	1.03 (−0.46, 2.53)	1.08 (−0.47, 2.64)

**Table A3:** Results of sensitivity analyses by changing the model options for the crossbasis function of the daily mean temperature on the association between TV, and cardiovascular and respiratory disease hospital admissions on all exposure days for all ages. The results were scaled per IQR increase in TV.

	Percent Increase in Cardiovascular Disease Hospitalisations (%)						
	Exposure Days						
**Variable**	**0–1**	**0–2**	**0–3**	**0–4**	**0–5**	**0–6**	**0–7**
**Degrees of Freedom for Natural Spline of Temperature**							
4	2.21 (0.57, 3.88)	2.74 (1.06, 4.44)	2.55 (0.92, 4.20)	2.24 (0.61, 3.89)	2.51 (0.90, 4.16)	2.24 (0.64, 3.87)	2.19 (0.52, 3.89)
5	2.21 (0.57, 3.88)	2.74 (1.06, 4.44)	2.55 (0.92, 4.20)	2.24 (0.61, 3.89)	2.51 (0.90, 4.16)	2.24 (0.64, 3.87)	2.19 (0.52, 3.89)
6	2.21 (0.57, 3.88)	2.74 (1.06, 4.44)	2.55 (0.92, 4.20)	2.24 (0.61, 3.89)	2.51 (0.90, 4.16)	2.24 (0.64, 3.87)	2.19 (0.52, 3.89)
Maximum lag (28)	2.08 (0.44, 3.74)	2.62 (0.95, 4.31)	2.42 (0.80, 4.06)	2.10 (0.50, 3.72)	2.33 (0.75, 3.93)	2.04 (0.48, 3.63)	1.98(0.35, 3.64)
Tmin	2.64 (0.92, 4.39)	3.05 (1.41, 4.73)	2.94 (1.35, 4.55)	2.67 (1.08, 4.29)	2.89 (1.31, 4.49)	2.54 (0.98, 4.12)	2.45 (0.83, 4.10)
Tmax	0.92 (−1.30, 3.20)	1.59 (−0.62, 3.86)	1.30 (−0.76, 3.40)	0.91 (−1.13, 2.99)	1.49 (−0.57, 3.60)	1.14 (−0.93, 3.24)	1.03 (−1.09, 3.19)
	**Percent increase in respiratory disease hospitalisations (%)**						
**Variable**	**0–1**	**0–2**	**0–3**	**0–4**	**0–5**	**0–6**	**0–7**
**Degrees of freedom for natural spline of temperature**							
4	2.77 (1.21, 4.36)	2.43 (0.84, 4.05)	1.14 (−0.40, 2.70)	1.33 (−0.21, 2.90)	1.17 (−0.36, 2.72)	0.87 (−0.64, 2.40)	0.89 (−0.68, 2.49)
5	2.77 (1.21, 4.36)	2.43 (0.84, 4.05)	1.14 (−0.40, 2.70)	1.33 (−0.21, 2.90)	1.17 (−0.36, 2.72)	0.87 (−0.64, 2.40)	0.89 (−0.68, 2.49)
6	2.77 (1.21, 4.36)	2.43 (0.84, 4.05)	1.14 (−0.40, 2.70)	1.33 (−0.21, 2.90)	1.17 (−0.36, 2.72)	0.87 (−0.64, 2.40)	0.89 (−0.68, 2.49)
Maximum lag (21)	2.62 (1.06, 4.21)	2.26 (0.67, 3.87)	0.95 (−0.57, 2.50)	1.12 (−0.39, 2.66)	0.98 (−0.51, 2.49)	0.68 (−0.79, 2.17)	0.70 (−0.83, 2.26)
Tmin	2.43 (0.77, 4.11)	2.12 (0.54, 3.73)	1.07 (−0.44, 2.60)	1.41 (−0.11, 2.95)	1.28 (−0.21, 2.80)	0.94 (−0.54, 2.43)	0.89 (−0.64, 2.45)
Tmax	2.57 (0.42, 4.76)	1.50 (−0.61, 3.66)	−0.77 (−2.71, 1.21)	−0.28 (−2.22, 1.69)	−0.32 (−2.27, 1.67)	−0.62 (−2.56, 1.35)	−0.47 (−2.46, 1.57)

**Table A4:** Results of sensitivity analyses by changing the model options for relative humidity on the association between TV, and cardiovascular and respiratory disease hospital admissions on all exposure days for all ages. The results were scaled per IQR increase in TV.

	Percent Increase in Cardiovascular Disease Hospitalisations (%)						
	Exposure days						
**Variable**	**0–1**	**0–2**	**0–3**	**0–4**	**0–5**	**0–6**	**0–7**
**Degrees of Freedom for Natural Spline of Relative Humidity**							
3	2.21 (0.57, 3.88)	2.74 (1.06, 4.44)	2.55 (0.92, 4.20)	2.24 (0.61, 3.89)	.51 (0.90, 4.16)	2.24 (0.64, 3.87)	2.19 (0.52, 3.89)
4	2.20 (0.56, 3.87)	2.72 (1.05, 4.43)	2.52 (0.89, 4.18)	2.21 (0.58, 3.86)	2.49 (0.87, 4.14)	2.22 (0.61, 3.85)	2.17 (0.50, 3.87)
5	2.20 (0.56, 3.87)	2.72 (1.04, 4.42)	2.53 (0.90, 4.18)	2.21 (0.58, 3.87)	2.49 (0.87, 4.14)	2.22 (0.61, 3.85)	2.17 (0.49, 3.87)
6	2.25 (0.60, 3.91)	2.81 (1.13, 4.52)	2.58 (0.94, 4.23)	2.23 (0.60, 3.88)	2.53 (0.91, 4.18)	2.27 (0.66, 3.91)	2.23 (0.55, 3.93)
RH	2.31 (0.69, 3.95)	2.81 (1.14, 4.50)	2.60 (0.98, 4.25)	2.29 (0.67, 3.94)	2.58 (0.97, 4.22)	2.31 (0.71, 3.93)	2.26 (0.59, 3.95)
RH_cat	2.24 (0.60, 3.90)	2.74 (1.07, 4.44)	2.54 (0.92, 4.20)	2.23 (0.60, 3.88)	2.52 (0.90, 4.16)	2.24 (0.63, 3.87)	2.18 (0.51, 3.88)
	**Percent increase in respiratory disease hospitalisations (%)**						
**Variable**	**0–1**	**0–2**	**0–3**	**0–4**	**0–5**	**0–6**	**0–7**
**Degrees of Freedom for Natural Spline of Relative Humidity**							
3	2.77 (1.21, 4.36)	2.43 (0.84, 4.05)	1.14 (−0.40, 2.70)	1.33 (−0.21, 2.90)	1.17 (−0.36, 2.72)	0.87 (−0.64, 2.40)	0.89 (−0.68, 2.49)
4	2.77 (1.21, 4.36)	2.43 (0.84, 4.05)	1.15 (−0.39, 2.71)	1.34 (−0.20, 2.91)	1.18 (−0.35, 2.73)	0.88 (−0.64, 2.41)	0.90 (−0.67, 2.49)
5	2.76 (1.20, 4.35)	2.42 (0.83, 4.04)	1.14 (−0.40, 2.70)	1.33 (−0.22, 2.90)	1.16 (−0.36, 2.71)	0.86 (−0.65, 2.40)	0.88 (−0.69, 2.48)
6	2.75 (1.18, 4.34)	2.40 (0.81, 4.02)	1.12 (−0.42, 2.69)	1.32 (−0.22, 2.89)	1.15 (−0.37, 2.70)	0.85 (−0.66, 2.38)	0.87 (−0.70, 2.46)
RH	2.93 (1.38, 4.49)	2.57(0.98, 4.17)	1.26 (−0.27, 2.82)	1.44 (−0.10, 3.00)	1.28 (−0.24, 2.82)	0.97 (−0.53, 2.50)	1.00 (−0.57, 2.59)
RH_cat	2.83 (1.27, 4.41)	2.49 (0.90, 4.10)	1.19 (−0.35, 2.75)	1.36 (−0.18, 2.93)	1.20 (−0.32, 2.75)	.89 (−0.62, 2.42)	0.91 (−0.66, 2.50)
